# Hyponatremia and stroke mimic: a case report

**DOI:** 10.11604/pamj.2022.43.39.36701

**Published:** 2022-09-23

**Authors:** Pharan Djimafo Tiwet, Isabelle Tchounkeu Kuigwa, Reine Sandrine Mendeuka, Marie-Dominique Gazagnes

**Affiliations:** 1 Critical Care Department, Brugmann University Hospital, *Université Libre de Bruxelles, Brussels, Belgium*; 2 Emergency Department, Brugmann University Hospital, *Université Libre de Bruxelles*, Brussels, Belgium,; 3 Neurology Department, Brugmann University Hospital, *Université Libre de Bruxelles*, Brussels, Belgium

**Keywords:** Hyponatremia, acute stroke, stroke mimic, Schwartz-Bartter syndrome, case report

## Abstract

The differential diagnosis of acute stroke is often a challenge for the emergency care staff. In medical literature, about 30% of patients presenting in an emergency department with suspected stroke at initial assessment are actually stroke mimic. We report here a case of a 61-year-old woman who got admitted at the emergency service for an acute stroke that was actually a symptomatic hyponatremia due to a Schwartz-Bartter syndrome associated with an undiagnosed breast cancer. It is very important to quickly identify and manage patients presenting with a high probability of acute stroke even though many other pathological conditions can present with the same clinical onset. Electrolyte disturbances are one of those stroke mimic conditions that are usually reversible if properly identified and treated on time.

## Introduction

Acute stroke is the major cause of disability. It requires immediate assessment and treatment. Stroke can occur with a rich pattern of symptoms that are characteristic but non specific. There are many other clinical conditions that can present with stroke-alike features [1]. Stroke mimic is a medical condition presenting with an acute neurological deficit that simulates real stroke. Metabolic disorders such as hyponatremia which is the most common electrolyte disorder can cause a stroke mimic condition. We present a case of stroke mimic caused by severe hyponatremia.

## Patient and observation

**Patient information:** a 61-year-old woman was referred to the emergency department for sudden aphasia and right hemiparesis. The patient lived alone at home and her family noticed the abnormality approximately 13 hours after the onset and called for emergency assistance. She had a history of alcoholism, untreated hypertension and chronic obstructive pulmonary disease related to tobacco smoking. She did not have any home medication.

**Clinical findings:** by the time of admission, the patient presented a blood pressure of 190/70 mmHg, a heart rate of 114 beats/minute, an oxygen saturation of 94% on air, breathing at 15 times/minute. Her temperature was 38.1°C and her glycemia 117 mg/dL. Her neurological exam showed a limited fluency, a right hemiparesis with a strength loss evaluated at 3/5 (MRC scale) and a gaze deviation to the left. Plantar response was extensor on the right side, her Glascow Coma Score (GCS) was 14 (E4V4M6) due to confusion and the National Institute of Health Stroke score (NIHSS) was 15.

**Figure 1:**
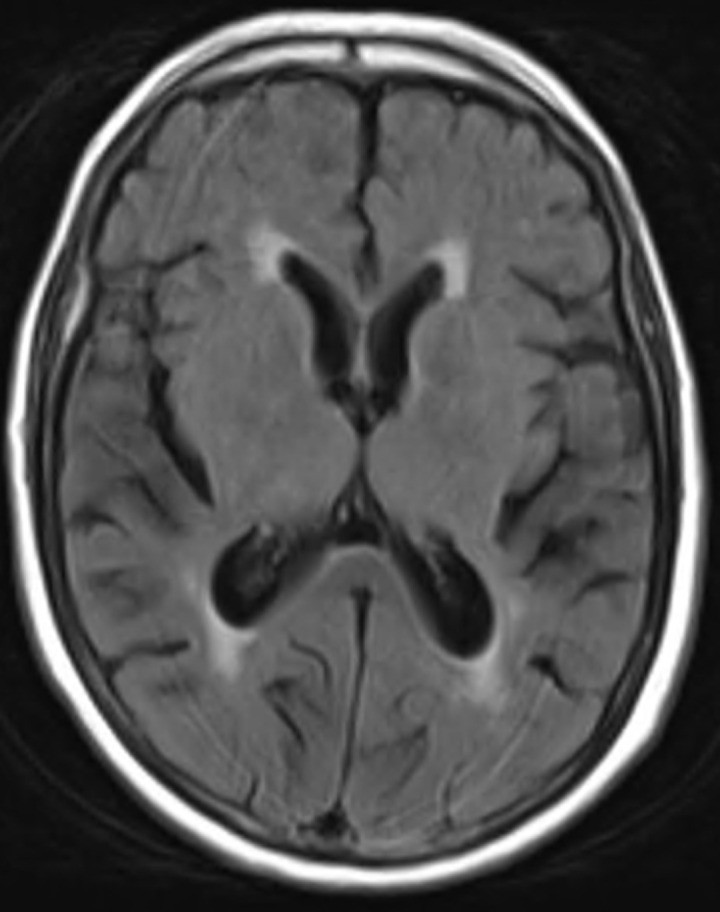
normal axial brain MRI serie 7 T2 Flair

**Timeline:** the day after her admission, the patient had no improvement in her clinical condition. The NIHSS score was unchanged and the follow-up brain CT scan performed after 12 hours showed no new lesion. On the second day, the patient was able to answer questions more appropriately than before, but still presented a gaze deviation and a motor deficit. The serum sodium was then 125 mmol/L under saline perfusion. The complete regression of symptoms was then observed on the third day with a natremia of 129 mmol/L. The brain magnetic resonance conducted the same day showed no acute ischemic lesion (Figure 1).

**Diagnostic assessment:** the Computed tomography (CT) angiography and CT perfusion of the brain conducted a few minutes after the admission of the patient at the emergency room did not demonstrate any acute lesion but only an old small-vessel disease lesion. The blood test performed on the admission showed a sodium level of 120 mmol/l, a plasma osmolality of 258 mosm/Kg, no inflammatory syndrome, no coagulation disorder and a normal renal function. The electrocardiogram demonstrated a sinus tachycardia. The electroencephalogram proceeded on the same day showed a moderate encephalopathy with left hemispheric hypovoltage and absence of no epileptic activity. Other complementary investigations conducted at admission were hemocultures, bacterial (aerobic and anaerobic) and viral serologies (Hepatitis B, Hepatitis C, Citomegalovirus, Herpes simplex, HIV) all resulted negative. The lumbar puncture was normal.

**Therapeutic intervention:** as the clinic of the patient remained unchanged, the high probability of acute ischemic stroke sustained a specific treatment with antiplatelet agents and lipid-lowering drugs. From her admission, the patient was no more in the thrombolysis criteria which was therefore not indicated. A perfusion of saline liquid was also started to correct the hyponatremia.

**Follow-up and outcomes:** the patient was discharged against medical advice on day 4, with complete resolution of her neurologic symptoms and a natremia of 130 mmol/L. The diagnosis of stroke mimic caused by hyponatremia was therefore retained. A series of additional investigations to determine the origin of the hyponatremia was conducted on ambulatory basis. The complementary investigations were a thoracic CT-scan, a whole body Pet-scan and a breast ultrasound followed by a biopsy and revealed an infiltrating breast carcinoma. The cause of hyponatremia was therefore more compatible with the presence of a Schwartz-Bartter syndrome associated with breast cancer.

**Patient perspective:** the patient has been managed by a multidisciplinary team made of oncologist, surgeon and radiotherapist. She had a neoadjuvant chemotherapy treatment followed by a total mastectomy and radiotherapy. The follow up and the efficiency of the treatment are still undergoing.

**Patient consent:** the patient has been informed that her clinical condition has highlighted our interest for scientific publication over the subject. The patient gave her consent and agreement for her clinical history and data to be used for this reporting.

## Discussion

In the literature, about 30% of patients presenting in the emergency department with high probability of acute stroke at initial assessment are actually stroke mimics [2,3]. Stroke mimic is a medical condition presenting with acute neurological symptoms simulating a real acute stroke [4]. Common mimic stroke causes are seizures, metabolic disorders (such as electrolytes disturbances, glycemic disorders…), migraine, sepsis, mass lesion and psychogenic disorders [5,6].

Metabolic disorders represent the second cause of stroke mimic and the most frequent electrolyte disorder encountered in stroke mimic is hyponatremia. About 21% of patients with severe hyponatremia manifest with neurological features such as confusion, abnormal consciousness but also focal deficiency, even if it is unusual [7]. Hyponatremia is defined as a serum sodium concentration below 135 mmol/L. It is classified into hypotonic or non-hypotonic hyponatremia. Non-hypotonic hyponatremia is usually caused by hyperglycemia but also by mannitol or hypertonic radiocontrast product administration and pseudohyponatremia (which is due to a high concentration of triglyceride, cholesterol, or proteins).

**Figure 2:**
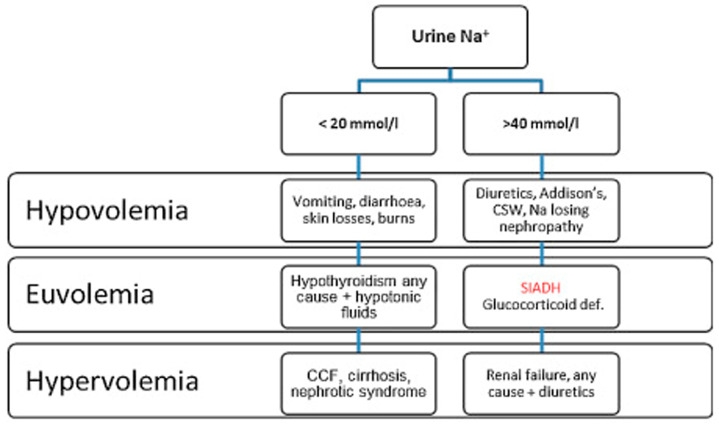
diagnosis of hyponatremia

Hypotonic hyponatremia is distinguished into hypovolemic, euvolemic and hypervolemic based on the clinical features of the patient. Hypovolemic hyponatremia is defined as a reduction of total body water with decrease of total body sodium usually caused by vomiting, diarrhea and use of diuretic agents. Hypervolemic hyponatremia is frequent in patients with heart failure, liver failure and renal failure, due to an increased total body water. Euvolemic hyponatremia can occur in patients with hypothyroidism, adrenal insufficiency and also Schwartz-Bartter Syndrome (SBS). Common causes of SBS include tumors, medications, neurological and pulmonary diseases [8]. The Figure 2 (CCF: Congestive cardiac failure; CSW: Cerebral salt wasting syndrome; SIADH: Syndrome of inappropriate secretion of antidiuretic hormone) reports a simplified diagnostic algorithm of hyponatremia [9].

Severe hyponatremia disturbance is a stroke mimic condition that has already been described in literature but frequently underestimated or unconsidered by clinicians in the emergency department in charge of patients with a high probability of acute stroke. In our case, hyponatremia was caused by SBS in the context of an undiagnosed breast cancer. The presentation was clearly acute stroke-alike with clinical patterns that decreased progressively with the correction of the sodium disorder until a total recovery after three days [10].

## Conclusion

Although stroke mimic is most of the time a reversible condition when the cause is identified and treated, the clinical presentation of acute stroke leads to an urgent management and treatment of the patient considering how high the disability outcome can be. Nevertheless, it is also important to remember the numerous conditions mimicking an acute stroke such as electrolyte disorder which is reversible with a good outcome if corrected properly on time.
